# Neural correlates associated with processing food stimuli in anorexia nervosa and bulimia nervosa: an activation likelihood estimation meta-analysis of fMRI studies

**DOI:** 10.1007/s40519-022-01390-x

**Published:** 2022-03-19

**Authors:** Madeline Bronleigh, Oliver Baumann, Peta Stapleton

**Affiliations:** School of Psychology, Bond University, Gold Coast, QLD Australia

**Keywords:** fMRI, Eating disorder, Anorexia nervosa, Bulimia nervosa

## Abstract

**Purpose:**

Various neurobiological models have utilised symptom categories to explore the underlying neural correlates in both anorexia nervosa (AN) and bulimia nervosa (BN). The aim of this research was to investigate the brain activity patterns associated with viewing food stimuli in anorexia nervosa and bulimia nervosa.

**Methods:**

Electronic databases including PsycInfo and PubMed were systematically searched from data base inception until 1st of December 2020, identifying 14 suitable functional magnetic resonance imaging studies (fMRI), involving 470 participants. ALE meta-analysis was used to statistically analyse the overlap of activation foci from different fMRI studies in response to visual food stimuli.

**Results:**

Comparing patients with AN with healthy control (HC), we detected hypoactivation in brain areas related to reward processing (i.e., amygdala and lentiform nucleus), and interoceptive processing (i.e., insula). In addition, patients with AN showed hyperactivations in cognitive control areas (i.e., prefrontal and anterior cingulate cortex). In contrast, patients with BN exhibited hyperactivations in brain areas related to reward processing (i.e., lentiform nucleus), and interoceptive processing (i.e., insula). Furthermore, patients with BN showed hypoactivations in brain regions associated with cognitive control (i.e., prefrontal and anterior cingulate cortex).

**Conclusions:**

Our study shows differing neural endotypes of the two types of eating disorders, that underpin their behavioural phenotypes. While exploratory in nature, these findings might be relevant for guiding new emerging therapies, including invasive and non-invasive neuromodulation techniques in treatment of eating disorders.

**Level of evidence:**

Level I, meta-analysis.

## Introduction

Anorexia nervosa (AN) and bulimia nervosa (BN) are psychiatric disorders with a very high burden of disease. Eating disorder patients commonly suffer several medical complications involving endocrine, cardiovascular, gastrointestinal, renal and neurological systems [46]. Consequently, eating disorders are associated with increased rates of mortality compared with the general population [46], and individuals with AN have the highest mortality rates of all other psychiatric disorders [33, 45].

AN and BN feature overlapping (such as fixation on weight and appearance) as well as distinct symptoms (such as extreme restriction of calorie intake vs. binging and purging) [1]. While several factors including sociocultural, psychological and biological aspects have been suggested to contribute to the development and maintenance of these eating disorders, the aetiology is still inconclusive [45].

Analogous with several other psychiatric illnesses, there has been increasing research surrounding the underlying neurobiological links associated with the contribution and maintenance of eating disorders [31]. Various neurobiological models have utilised symptom categories to explore the underlying neural correlates, both AN and BN share and what contributes to their differing clinical presentations [31]. For example, food restriction is a key characteristic of AN, and imbalances in the brain systems involved in emotion, reward processes and decision-making processes, have been implicated in AN presentation [28]. By contrast, high levels of impulsivity and a lack of inhibition-control leading to binge-eating can be demonstrated in BN, and differences in these neural correlates compared to AN have been suggested [31, 60].

Reward processing has been proposed to have an integral role in accounting for extreme differences in food intake between individuals presenting with eating disorders [23]. Individuals with anorexia commonly report anhedonia, an impaired ability to derive pleasure from usually enjoyable activities [38]. Instead, food restriction is thought to be perceived as initially rewarding, due to weight-related cognitions, and subsequently, sustained due to the conditioning of the reward [28, 56]. Reduced reward sensitivity has been demonstrated in individuals with AN, with an increased ability to delay monetary rewards for example [28]. In contrast, individuals with BN report increased novelty seeking and reward sensitivity [23]. The complex neural mechanisms underlying reward processing employ multiple brain regions [32] to perform a series of sub-processes, including reward anticipation reward association, re-appraisal of reward value, and reward-seeking behaviour regulation. Key areas are the striatum, predominantly the including the nucleus accumbens (NAc) [22], as well as the amygdala [47].

Second, interoceptive processing refers to the perception of one’s physiological functions, such as fullness, hunger, and taste [57]. Interoception centers on the anterior insula, through which the peripheral nervous system communicates to the limbic and cortical control centres of homeostasis [7, 30]. Due to individuals with eating disorders displaying abnormal perceptions of hunger, theories have suggested that impaired interoceptive processing has contributed to the maintenance of their disorders [9, 18, 28, 55].

Finally, cognitive control regions are interconnected with both, reward-related regions and areas involved in interoception, and are considered to modulate eating behavior [22, 32, 46]. Chiefly, the prefrontal cortex, is thought to integrate goals, affective valence, interoceptive states, and sensory input to guide control functions, such as behavioural inhibition, planning and decision making [21, 22, 41, 47, 65]. Furthermore, the anterior cingulate cortex performs additional executive functions associated with affect-value attribution, emotional regulation, and conflict monitoring [64]. Finally, the orbitofrontal cortex aids in subjective value attribution and decision making [22, 32].

## Using neuroimaging to investigate the mechanisms of eating disorders

Recent advances in neuroimaging techniques have allowed for an increase in research surrounding the underlying neural mechanisms associated with the initiation and maintenance of eating disorders [31]. Especially, functional magnetic resonance imaging (fMRI), the most popular neuroimaging tool, has led to substantial advancements in the understanding of neurobiological models of eating disorders [17].

While the use of fMRI studies to identify neural regions associated with eating disorders has been undoubtedly advantageous, the findings are inherently variable. fMRI studies often use relatively small sample sizes, which can lead to issues with replicability and false positive results [9, 17, 51]. Therefore, inconsistencies between existing fMRI studies investigating eating disorders are not uncommon, which makes drawing conclusion about the neurobiological underpinnings problematic [17].

To address these limitations, coordinate-based meta-analysis can be used. This method statistically establishes concurrence across fMRI studies by pooling activation data [12, 51]. Pooling data across similar studies allows for increased statistical power, determines inter-study trends, and enables findings occurring by chance to be separated from the consistent results [51]. One of the most widely used tools for coordinate-based meta-analysis is Activation Likelihood Estimation (ALE) [10, 51, 58].

### Current study

The aim of this study was to conduct a systematic ALE meta-analysis of existing fMRI studies to explore the commonalities and differences in brain activation patterns associated with AN and BN, relative to healthy controls (HC). Given the relevance of food for eating disorders, this review focused on task-based fMRI studies that required participants to observe food stimuli.

### Hypotheses

1. We hypothesised that when looking at food stimuli, AN patients would show hypoactivity (i.e., reduced activity) in the brain regions related to reward processing, relative to HC. In contrast, we predicted BN patients to show hyperactivity (i.e., increased activity), relative to HC.
2. We hypothesised that when looking at food stimuli, both AN and BN patients would exhibit abnormal activity, relative to HC, in brain regions related to interoceptive processing.
3. We hypothesised that patients with AN would show hyperactivity in brain regions related to cognitive control, while individuals with BN would show hypoactivity, both relative to HC.

## Method

### Literature search and article selection

**Fig. 1:**
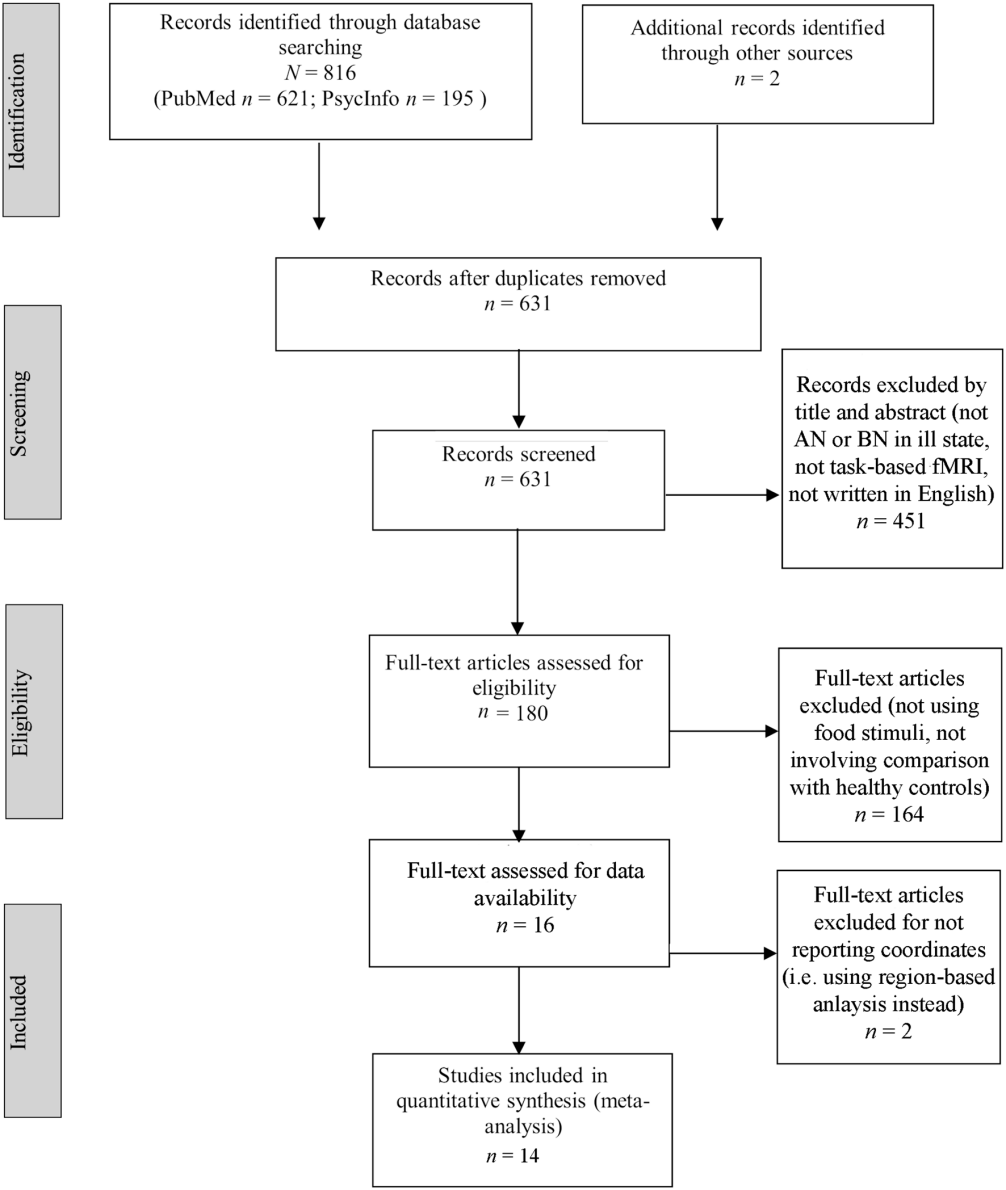
Prisma flowchart of search and selection procedure

The review was conducted in accordance with the Preferred Reporting Items fo Systematic Reviews and Meta-Analysis (PRISMA) guidelines [42] (see Fig. 1).

### Search strategy, eligibility criteria and data extraction

The electronic databases employed for the search included PsycInfo and PubMed. To identify additional relevant studies, manual searches within the reference lists of examined studies were conducted. All relevant published journal articles, with no year limits were included in the search. The key terms used were (“fMRI” OR “functional magnetic resonance imaging”) AND (“Anorexia Nervosa” OR “Bulimia” OR "Eating Disorder”). After screening for duplicates, the titles and abstracts and of 631 studies were independently assessed by two reviewers for the following criteria: (a) involving patients with AN or BN in ill state (i.e., not recovered); (b) using task-based fMRI (i.e., no resting-state studies); (c) written in English. This initial screening stage led to the rejection of 451 articles. The full-text of the remaining 180 articles were independently assessed by two reviewers for the following additional criteria: (e) using tasks focused on food stimuli; (f) involving a statistical contrast of a patient sample (AN or BN) relative to a HC sample. This led to the exclusion of a further 164 articles. Finally, two articles were excluded for not reporting activation coordinates (i.e., those two studies used an region of interest-based approach). The final sample of studies comprised, therefore, 14 studies (see Table 1). From those studies two reviewers independently extracted the type of disorder (i.e., AN, BN, and HC), the sample size, the tasks performed, and the fMRI activation coordinates.

**Table 1:** Studies included ALE meta-analysis

Study	Participants	AN Subtype	Duration of Illness	Age	BMI	MRI Field Strength (Tesla)	Task
Bohon and Stice (2011)	13 with BN 13 HC		Not stated	20.3 20.3	23.9 23.2	3	Viewing a glass of chocolate milkshake vs. a glass of water
Brooks et al. (2011)	8 with BN 24 HC		12 years	25 26	21.6 21.7	1.5	Viewing a wide range of food vs. non-food stimuli
Brooks et al. (2012)	18 with AN 24 HC	11 rAN, 7 bpAN,	7.2 years	26 26	15.7 21.7	1.5	Viewing a wide range of food vs. non-food stimuli
Holsen et al. (2012)	12 with AN 11 HC	12 rAN	5 years	21.8 21.6	18* 22.4	3	Viewing a wide range of food vs. non-food stimuli
Horndasch et al. (2018)	16 with AN 16 HC	8 rAN, 7 bpAN, 1 atypAN	8.7 years	26.7 26.8	16.2 21.4	3	Viewing a wide range of food vs. non-food stimuli
Joos et al. (2011)	11 with AN 11 HC	11 rAN	5 years	25 26	16.2 21.1	3	Viewing savory and sweet food vs. non-food stimuli
Kim et al. (2012)	18 with AN 20 with BN 20 HC	6 rAN, 12 bpAN	3.8 years 3.8 years	25.2 22.9 23.3	16 21.6 19.9	1.5	Viewing high-calorie food vs. non-food stimuli
Lee et al. (2017)	12 with BN 14 HC		7.5 years	23.7 23.3	21.5 20.4	3	Viewing a wide range of food vs. non-food stimuli
Santel et al. (2006)	13 with AN 10 HC	13 rAN	0.8 years	16.1	16 20.5	1.5	Viewing high-calorie savory and sweet food vs. non-food stimuli
Scaife et al. (2016)	12 with AN 16 HC	12 rAN	10.3 years	29.4 24.3	15.4 21.2	3	Viewing of high calorie vs. low calorie food stimuli
Schienle et al. (2009)	14 with BN 19 HC		7.3 years	23.1 22.3	22.1 21.7	1.5	Viewing a wide range of food vs. non-food stimuli
Uher et al. (2004)	16 with AN 10 with BN 19 HC	9 rAN, 7 bpAN	13.1 years 14.8 years	26.9 29.8 26.7	16 22.4 22.4	1.5	Viewing savory and sweet food vs. non-food stimuli
Van den Eynde et al. (2013)	21 with BN 23 HC		Predominately in the 5–10 years range	28 27.3	23.4 21.3	1.5	Viewing savory and sweet food vs. non-food stimuli
Young et al. (2020)	16 with AN 20 HC	Not specified	15.4 years	31.4 26.7	15.9 21.2	1.5	Viewing a wide range of food vs. non-food stimuli

The Holsen et al. (2012) study used the DSM-IV weight criterion for AN (i.e., < 85th percentile of normal body weight). According to the ICD-10 criterion for AN (BMI ≤ 17.5), some patients would be considered weight restored

### Activation likelihood estimation

To identify the commonalities and differences in brain pattern associated with AN and BN, an activation likelihood estimation (ALE) analysis was performed, using GingerALE software Version 3.0.2 [10, 12, 58]. ALE is a method for conducting coordinate-based meta-analysis to statically analyse the overlap of activation foci from different fMRI studies [10]. For each included study, the software generates a model activation (MA) map by merging each study’s total foci coordinates (*x*, *y*, *z*). Individual MA maps are then combined across studies to form an ALE map. This ALE map provides a statistical map of consistent activations across studies [10, 51]. To account for risk of bias, GingerALE applies an uncertainty of random effects model. To account for the natural variability across participants spatial smoothing is also applied. That is, the analysis models foci as Gaussian probability distributions, using a Full-Width-Half-Maximum (FWHM), allowing for the smoothing of data over nearby voxels. The distribution width reflects study size, with larger sample sizes weighted more strongly than smaller sample sizes [10, 12]. To control for the problem of multiple comparisons within the same voxel resulting in false positive clusters, a cluster level FWE threshold was employed [10]. Specifically, statistical contrasts were computed using a height threshold of *p* < 0.1 and a cluster-defining threshold of *p* < 0.05 (FWE corrected for multiple comparisons), based on 1000 permutations, which is considered an optimal thresholding level [10, 11]. To be able to combine and compare activation patterns form different studies it is necessary to use normalised data within the same stereotaxic space [35]. The icbm2tal conversation algorithm, as implemented in GingerALE, was used to convert any activation foci reported in Talairach space to MNI space [36]. To view and display the activation likelihood maps produced by GingerALE coordinates, Mango version 2.5 software was used (http://ric.uthscsa.edu/mango/).

### Statistical contrasts

To identify how brain patterns associated with viewing food images in AN and BN differ from those in HC, four between group contrast analyses were conducted. (1). Testing for hyperactivated brain regions when viewing food stimuli in AN relative to HC (AN > HC). (2). Testing for hypoactivated brain regions when viewing food stimuli in AN relative to HC (HC > AN). (3). Testing for hyperactivated brain regions when viewing food stimuli in BN relative to HC (BN > HC). (4). Testing for hypoactivated brain regions when viewing food stimuli in BN relative to HC (HC > BN).

## Results

### Anorexia nervosa vs. healthy controls

**Fig. 2:**
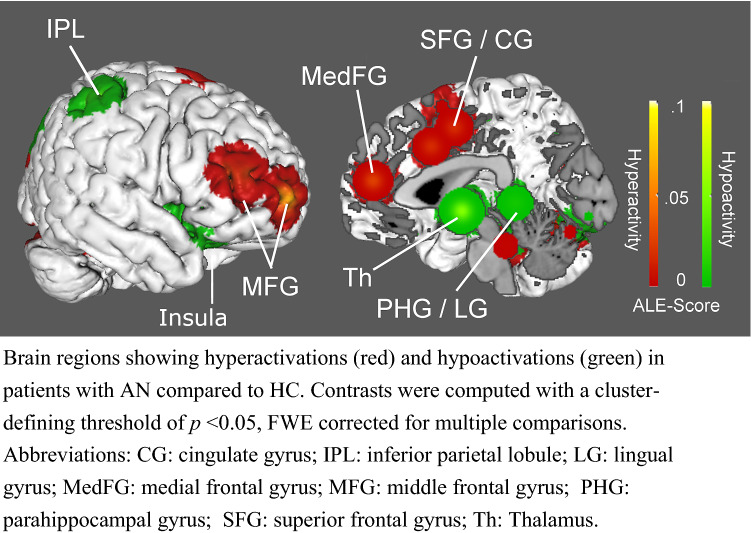
Hyperactivations and Hypoactivations in Patients in AN

The analysis was based on nine individual studies and revealed four clusters that showed significantly higher activity in AN patients relative to controls (i.e., hyperactivations related to viewing food images) that were located bilaterally in areas related to cognitive control (i.e., the frontal and cingulate cortex), as well as cerebellum (see Fig. 2 and Table 3). The reverse comparison revealed 11 clusters that showed significantly lower activity in anorexia patients compared to HC (i.e., hypoactivations related to viewing food images). Hypoactivated brain areas included those related to reward processing (i.e., amygdala and striatum) and interoceptive processing (i.e., insula). Additional areas of hypoactivations included the parietal cortex, cerebellum, thalamus, parahippocampal gyrus and lingual gyrus (see Fig. 2, and Table 2).

**Table 2:** AN hyperactivations and hypoactivation relative to HC

Region	Hemisphere	BA	MNI coordinates			ALE	*P*	*Z*	Cluster size
			*x*	*y*	*z*				Vol/mm^3^
AN > HC									
Cerebellum	R	–	14	− 74	− 16	7.672E-03	1.25E-04	3.663	68,432
Cerebellum	R	–	11	− 35	− 26	7.666E-03	1.25E-04	3.663	
Cerebellum	R	–	34	− 46	− 20	7.662E-03	1.28E-04	3.656	
Cerebellum	R	–	30	− 74	− 26	7.570E-03	1.37E-04	3.638	
Lingual gyrus	R	18	22	− 74	− 2	7.416E-03	1.58E-04	3.602	
Precuneus	R	31	32	− 76	24	6.862E-03	3.03E-04	3.429	
Medial frontal gyrus	L	9	8	47	12	6.967E-03	2.70E-04	3.460	38,704
Superior frontal gyrus	R	10	28	64	0	7.144E-03	1.97E-04	3.544	
Middle frontal gyrus	R	46	44	42	10	6.646E-03	5.13E-04	3.283	
Cingulate gyrus	L	24	− 0.3	6.3	46	3.60E-04	3.19E-02	1.853	32,784
Cerebellum	L	–	− 42	− 72	− 22	7.829E-03	3.02E-05	4.011	22,944
Cerebellum	L	–	− 18	− 74	− 24	7.592E-03	1.33E-04	3.646	
HC > AN									
Striatum	R	–	33	8	4	6.707E-03	2.52E-04	3.479	31,240
Inferior frontal gyrus	R	47	36	23	− 11	6.529E-03	3.73E-04	3.372	
Amygdala	L	–	− 21	− 10	− 11	6.519E-03	3.73E-04	3.372	30,656
Thalamus	L	–	− 3	− 7	− 5	6.336E-03	4.31E-04	3.332	
Lingual gyrus	R	18	14	− 86	− 6	6.618E-03	2.75E-04	3.455	17,200
Parahippocampal gyrus	L	30	− 9	− 40	1	6.519E-03	3.73E-04	3.372	16,776
Insula	L	–	− 30	17	7	6.519E-03	3.73E-04	3.372	16,768
Inferior parietal lobule	L	40	− 34	− 44	50	7.831E-03	8.12E-05	3.771	14,552
Cerebellum	L	–	− 32	− 78	− 20	7.958E-03	4.03E-05	3.942	14,480
Cerebellum	R	–	20	− 44	− 20	8.638E-03	9.62E-06	4.273	14,040
Insula	R	13	48	− 24	− 2	8.234E-03	1.58E-05	4.162	14,016
Inferior parietal lobule	R	40	49	− 31	48	7.876E-03	7.71E-05	3.784	13,344
Precuneus	R	19	35	− 73	36	7.876E-03	7.71E-05	3.784	13,240

Anatomical locations and spatial coordinates of ALE analysis showing significant activations (with a cluster-defining threshold of *p* < 0.05, FWE corrected for multiple comparisons)

*BA* brodmann area

Brain regions showing hyperactivations (red) and hypoactivations (green) in patients with AN compared to HC. Contrasts were computed with a cluster-defining threshold of *p* < 0.05, FWE corrected for multiple comparisons. Abbreviations: *CG* cingulate gyrus, *IPL* inferior parietal lobule, *LG* lingual gyrus, *MedFG* medial frontal gyrus, *MFG* middle frontal gyrus, *PHG* parahippocampal gyrus, *SFG* superior frontal gyrus, *Th* Thalamus.

### Bulimia nervosa vs. healthy controls

**Fig. 3:**
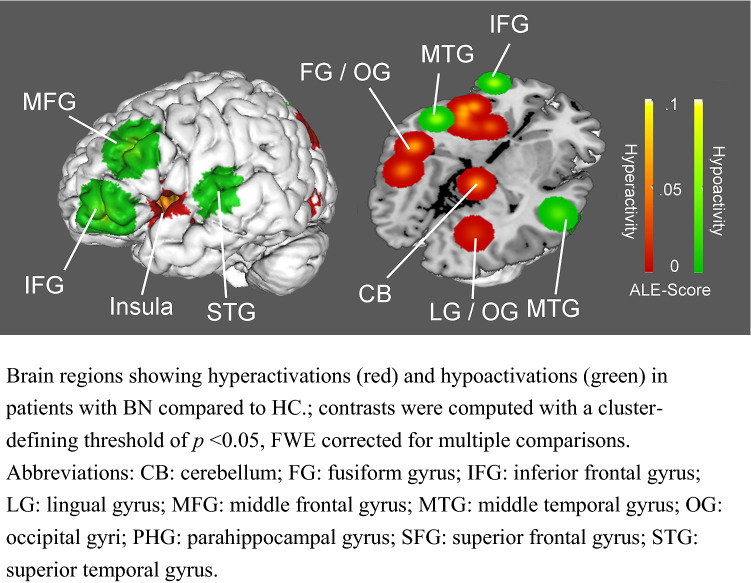
Hyperactivations and Hypoactivations in Patients in BN

The analysis was based on seven individual studies and revealed six clusters that showed significantly higher activity in BN patients relative to controls (i.e., hyperactivations related to viewing food images). Hyperactivated brain areas included those related reward processing (striatum), interoception (insula), and visual processing (cuneus, lingual and fusiform gyrus). In addition, there was significant hyperactivity in the bilateral cerebellum) (see Fig. 3 and Table 4). The reverse comparison revealed seven clusters that showed significantly lower activity in BN patients compared to HC (i.e., hypoactivations related to viewing food images). Hypoactivated brain areas included predominantly those related to cognitive control (prefrontal and cingulate cortex). Additional areas of hypoactivations included the precunues and temporal cortex (see Fig. 3, and Table 3).

**Table 3:** BN hyperactived and hypoactivated brain regions when looking at food images relative to HC

Region	Hemisphere	BA	MNI coordinates			ALE	*P*	*Z*	Cluster size
			*x*	*y*	*z*				Vol/mm^3^
BN > HC									
Insula	L	–	− 36	− 6	− 8	7.563E-03	9.06E-05	3.744	42,184
Insula	L	13	− 42	2	− 4	7.532E-03	9.16E-05	3.741	
Insula	L	–	− 38	0	4	7.374E-03	1.01E-04	3.716	
Striatum	L	–	− 26	− 4	2	7.369E-03	1.01E-04	3.716	
Insula	L	13	− 44	6	− 4	7.368E-03	1.01E-04	3.716	
Insula	L	13	− 40	12	− 4	7.361E-03	1.22E-04	3.669	
Striatum	L		− 18	4	− 10	7.361E-03	1.22E-04	3.669	
Middle temporal gyrus	L	37	− 48	− 44	− 4	7.144E-03	1.84E-04	3.562	22,272
Fusiform gyrus	L	37	− 40	− 64	− 8	6.444E-03	5.52E-04	3.263	
Cerebellum	R	–	4	− 42	− 8	7.876E-03	6.84E-05	3.814	21,424
Cerebellum	R	–	4	− 58	− 22	6.471E-03	5.46E-04	3.265	
Cuneus	L	17	− 22	− 80	12	8.430E-03	2.26E-05	4.080	21,040
Superior Occipital gyrus	L	19	− 34	− 72	34	7.144E-03	1.84E-04	3.562	
Anterior cingulate	R	32	12	42	21	7.331E-03	1.40E-04	3.633	18,504
Superior frontal gyrus	R	8	20	44	48	7.144E-03	1.84E-04	3.562	
Cuneus	R	17	24	− 80	12	8.371E-03	2.32E-05	4.073	18,072
Lingual gyrus	R	19	26	− 70	2	7.157E-03	1.55E-04	3.607	
HC > BN									
Middle frontal gyrus	L	10	− 44	44	− 8	7.951E-03	4.88E-05	3.896	14,464
Middle frontal gyrus	L	46	− 48	28	24	8.026E-03	2.99E-05	4.014	14,296
Precuneus	L	7	− 27	− 59	42	7.876E-03	7.88E-05	3.779	14,176
Precuneus	R	5	11	− 41	66	7.876E-03	7.88E-05	3.779	14,168
Middle temporal gyrus	R	21	58	− 26	− 10	8.453E-03	1.01E-05	4.263	13,976
Superior temporal gyrus	L	22	− 54	− 16	− 2	8.374E-03	1.40E-05	4.189	13,928
Middle temporal gyrus	R	39	48	− 68	30	8.482E-03	6.29E-06	4.367	11,216

Anatomical locations and spatial coordinates of ALE analysis showing significant activations (with a cluster-defining threshold of *p* < 0.05, FWE corrected for multiple comparisons)

*BA* brodmann area

Brain regions showing hyperactivations (red) and hypoactivations (green) in patients with BN compared to HC; contrasts were computed with a cluster-defining threshold of *p* < 0.05, FWE corrected for multiple comparisons. Abbreviations: *CB* cerebellum, *FG*,fusiform gyrus, *IFG* inferior frontal gyrus, *LG* lingual gyrus, *MFG* middle frontal gyrus, *MTG* middle temporal gyrus, *OG* occipital gyri, *PHG* parahippocampal gyrus, *SFG* superior frontal gyrus, *STG* superior temporal gyrus.

## Discussion

Our first hypothesis was that patients with AN would show hypoactivity in the brain regions related to reward processing compared to HC. In contrast, BN patients were predicted to show hyperactivity in brain regions related to reward processing. The results of the ALE analysis supported both predictions, by showing decreased activity in amygdala and striatum for the anorexia group and increased striatal activity for the bulimia group. Second, we hypothesized that when looking at food stimuli, both AN and BN patients would exhibit abnormal activity, relative to HC, in brain regions related to interoceptive processing. Again, the results of the ALE analysis supported both predictions, showing decreased insula activity for the anorexia group, and increased activity for the bulimia group. Finally, we hypothesised that patients with AN would show hyperactivity in brain regions related to cognitive control, while individuals with BN would show hypoactivity, both relative to HC. Also here, the ALE analyses supported the predictions; the AN group showed increased activity in cognitive-control-related areas (i.e., prefrontal cortex) relative to the HC group, while the BC group exhibited an inverse pattern (i.e., decreased activity of the prefrontal cortex).

The first key finding of the ALE analysis was decreased activity in amygdala and striatum for the anorexia group and increased striatal activity for the bulimia group. The amygdala is known to be play a central role in emotional processing [44] and conditioned learning, such as punishment anticipation and reward learning [22, 32]. The decreased activity in the anorexia group is, therefore, likely indicative that food rewards are indeed less rewarding. These findings are also in line with fMRI studies focused on gustatory perception which observed reduced amygdala activation in AN patients in response to taste stimuli [43, 63].

In addition, we identified decreased activity in the striatum for the anorexia group and increased activity in the bulimia group. The activation cluster covered both, the ventral and dorsal striatum. The ventral striatum is well known for its role in reward-related behaviour [8], but also the dorsal striatum is known to respond to reward stimuli [2], even though it is better known for its role in habit learning [14]. In addition, the dorsal striatum had been found to mediate behaviours involving eating, particularly of highly palatable, high-calorie foods [19, 29]. As individuals with AN are known to typically avoid high-calorie foods, whereas bulimia patient embrace it [20, 29], this in line with the differential brain activity patterns observed in this study.

Distinctions between the two patient groups in terms of reward processing are also indicated by differential activity patterns in visual cortex, i.e., we found decreased activity in anorexia patients and increased activity in bulimia patients, suggesting different degrees of visual attention being paid to food stimuli. Decreased activation in visual processing areas found in AN subjects may indicate they are employing cognitive strategies to show less attention towards the stimuli to support and maintain the anorexic state [62]. In contrast, BN individuals’ increased engagement towards the food stimuli, together with increased reward processing brain regions, suggests they display a heightened reward sensitivity towards food [13].

The second key finding of our analysis was decreased insula activity for the anorexia group, and increased activity for the bulimia group. Those activity patterns suggest that higher activity relates to greater interoceptive responsiveness, such as increased feelings of hunger or appetite in bulimia patients [34]. In contrast, the lower-than-normal activity in the anorexia group suggests, therefore, less interoceptive responsiveness to food stimuli [7], and potentially impaired ability to recognise internal hunger cues [7, 34].

Finally, our prediction of brain activity patterns indicative of lower-than-normal cognitive control in the bulimia group, and increased cognitive control in the anorexia group were supported by the ALE results. More specifically, the AN group displayed hyperactivity in the prefrontal cortex, inclusion specifically medial and lateral prefrontal areas that form part of the cognitive control network, governing effortful regulation of affective valence, selective attention, and inhibition control [22, 64, 66]. Increased activity of this network in the AN group is likely to represent a compensatory attempt to exert cognitive control over emotional and reward responses from food stimuli. Overall, these findings suggest an increased responsiveness in terms of monitoring and regulating emotions and food-related motivations, which subsequently lead to restrictive appetitive behavioural responses commonly seen in individuals with AN [17, 27, 28, 55].

In contrast, BN patients displayed hypoactivity in the prefrontal control network, suggesting a decreased responsiveness in terms of monitoring and modulating the emotions and reward-related motivations that the food stimuli may induce [65]. Therefore, this indicates that individuals with BN have less effective cognitive control, thus allowing for their heightened appetitive and reward systems to impinge, eventually resulting in binge eating behaviours [65, 67]. Our observations are also in line with studies showing reduced prefrontal activity in individuals with BN during tasks that require (non-food-related) response inhibition tasks [39, 40]. Furthermore, repetitive transcranial magnetic stimulation (rTMS) of the prefrontal cortex has been found to reduce food cravings in women prone to strong food cravings [59] and to binge eating [60].

Interestingly, both AN and BN patients showed hyperactivity in the cerebellum relative to HC. While not directly related to our hypotheses, this finding indicates a functional role for the cerebellum in the control of feeding behaviour. A cluster of nuclei within the cerebellum have been previously shown to have an inhibitory effect on glucose-sensitive neurons within the hypothalamus, suggesting a role in blood glucose homeostasis through regulation of food intake [69]. Another explanation may lie in the emerging evidence of a role of the cerebellum in the perception and regulation of emotions through its connectivity with the limbic networks [3].

### Strength and limits

To allow for valid conclusions, quantitative meta-analysis require a high degree comparability and constancy across the included studies. We, therefore, restricted the analysis on studies that involve viewing of food stimuli. While this ensures a high degree of internal consistency and validity it also limits the generalizability of the findings. For instance, our study does not provide insights into other facets of eating disorder, such as those related to body image. Our stringent exclusion criteria also resulted in a relatively low number of included studies (i.e., 14 studies, involving 470 participants) and the results should be, therefore, considered explorative in nature. It is important to note though that pooling data across a relatively small number of studies still has considerable advantages compared to results based on single studies. The evidence base in our analysis was also still sufficiently large to allow for stringent statistical thresholding (i.e., FWE correction), which provides a high level of confidence in the reliability of the results. A larger sample of studies would, however, also permit assessing effects of duration of illness on brain activation patterns. Structural imaging work has indicated that prolonged AN is associated with atrophy specifically in the cerebellum [15]. Nevertheless, in the current study, the typical duration of illness was comparable for the two disorders (Median_AN_ = 7.2 years; Median_BN_ = 7.5 years) and is, therefore, not a probable source of bias.

Another limitation to this study was that the AN sample was not split by restricting and binge-purge subtypes. While the minimum severity BMI criteria is still required for both subtypes of AN [1], it has been argued that the clinical features of AN binge-purge subtype may align more with BN symptomology in relation to eating behaviours [28, 31]. While many of the included studies in this meta-analysis reported the importance of considering AN by subtypes, the majority of these studies subsequently did not split their data by subtypes, due to limitations of small sample sizes [9, 16, 51].

Finally, while ALE is the most commonly meta-analytical tool for fMRI data, it is not the only approach. Seed-Based Analysis (also known as SDM) is an alternative approach, which in contrast to ALE takes also the effect size of included studies into consideration [48–50]. It is important to note that differences in meta-analytical approaches might lead to some degree of variability in the outcomes.

Despite this limitation our study provides a valuable summative assessment of the distinct the neural mechanisms underpinning eating disorders, which in turn might guide targeted future interventions, such as neuromodulation approaches treatments [60].

## What is already know on the subject?

Several existing fMRI studies have investigated the neural mechanisms underpinning AN and BN. There is, however, a large degree of variability among the results.

## What your study adds?

Using an ALE meta-analysis approach, allowed us to identify unique as well as overlapping brain activity for AN and BN relative to HC.

## Data Availability

The ALE cordantes are available via OSF: https://osf.io/4stf8/.

## References

[1] American Psychiatric Association. (2013). Diagnostic and statistical manual of mental disorders (5th ed).10.1176/appi.books.9780890425596

[2] Apicella P, Ljungberg T, Scarnati E, Schultz W (1991). Responses to reward in monkey dorsal and ventral striatum. Exp Brain Res.

[3] Baumann O, Mattingley JB (2012). Functional topography of primary emotion processing in the human cerebellum. Neuroimage.

[4] Bohon C, Stice E (2011). Reward abnormalities among women with full and subthreshold bulimia nervosa: a functional magnetic resonance imaging study. Int J Eat Disord.

[5] Brooks SJ, O'Daly O, Uher R, Friederich HC, Giampietro V, Brammer M, Williams SC, Schiöth HB, Treasure J, Campbell IC (2012). Thinking about eating food activates visual cortex with reduced bilateral cerebellar activation in females with anorexia nervosa: an fMRI study. PLoS ONE.

[6] Brooks SJ, O’Daly OG, Uher R, Friederich HC, Giampietro V, Brammer M, Williams SCR, Schiöth HB, Treasure J, Campbell IC (2011). Differential neural responses to food images in women with bulimia versus anorexia nervosa. PLoS One.

[7] Craig AD (2002). How do you feel? Interoception: the sense of the physiological condition of the body. Nat Rev Neurosci.

[8] Daniel R, Pollmann S (2014). A universal role of the ventral striatum in reward-based learning: evidence from human studies. Neurobiol Learn Mem.

[9] Donnelly B, Touyz S, Hay P, Burton A, Russell J, Caterson I (2018). Neuroimaging in bulimia nervosa and binge eating disorder: a systematic review. J Eat Disord.

[10] Eickhoff SB, Bzdok D, Laird AR, Kurth F, Fox PT (2012). Activation likelihood estimation meta-analysis revisited. Neuroimage.

[11] Eickhoff SB, Laird AR, Fox PM, Lancaster JL, Fox PT (2017). Implementation errors in the GingerALE software: description and recommendations. Hum Brain Mapp.

[12] Eickhoff SB, Laird AR, Grefkes C, Wang LE, Zilles K, Fox PT (2009). Coordinate-based activation likelihood estimation meta-analysis of neuroimaging data: a random-effects approach based on empirical estimates of spatial uncertainty. Hum Brain Mapp.

[13] Farmer RF, Nash HM, Field CE (2001). Disordered eating behaviors and reward sensitivity. J Behav Ther Exp Psychiatry.

[14] Featherstone RE, McDonald RJ (2004). Dorsal striatum and stimulus-response learning: lesions of the dorsolateral, but not dorsomedial, striatum impair acquisition of a simple discrimination task. Behav Brain Res.

[15] Fonville L, Giampietro V, Williams SC, Simmons A, Tchanturia K (2014). Alterations in brain structure in adults with anorexia nervosa and the impact of illness duration. Psychol Med.

[16] Frank GKW (2015). What causes eating disorders, and what do they cause?. Biol Psychiat.

[17] Frank GKW, Favaro A, Marsh R, Ehrlich S, Lawson EA (2018). Toward valid and reliable brain imaging results in eating disorders. Int J Eat Disord.

[18] Friederich H-C, Brooks S, Uher R, Campbell IC, Giampietro V, Brammer M, Williams SCR, Herzog W, Treasure J (2010). Neural correlates of body dissatisfaction in anorexia nervosa. Neuropsychologia.

[19] Fudge JL, Breitbart MA, Danish M, Pannoni V (2005). Insular and gustatory inputs to the caudal ventral striatum in primates. J Comp Neurol(1911).

[20] Fuglset TS, Landrø NI, Reas DL, Rø Ø (2016). Functional brain alterations in anorexia nervosa: a scoping review. J Eat Disord.

[21] Funahashi S (2017). Working memory in the prefrontal cortex. Brain Sci.

[22] Haber SN, Knutson B (2009). The reward circuit: linking primate anatomy and human imaging. Neuropsychopharmacology.

[23] Harrison A, Apos Brien N, Lopez C, Treasure J (2010). Sensitivity to reward and punishment in eating disorders. Psychiatry research.

[24] Holsen LM, Lawson EA, Blum J, Ko E, Makris N, Fazeli PK, Klibanski A, Goldstein JM (2012). Food motivation circuitry hypoactivation related to hedonic and nonhedonic aspects of hunger and satiety in women with active anorexia nervosa and weight-restored women with anorexia nervosa. J Psychiatry Neurosci.

[25] Horndasch S, Roesch J, Forster C, Dörfler A, Lindsiepe S, Heinrich H, Graap H, Moll GH, Kratz O (2018). Neural processing of food and emotional stimuli in adolescent and adult anorexia nervosa patients. PLoS ONE.

[26] Joos AA, Saum B, van Elst LT, Perlov E, Glauche V, Hartmann A, Freyer T, Tüscher O, Zeeck A (2011). Amygdala hyperreactivity in restrictive anorexia nervosa. Psychiatry Res.

[27] Kaye HW, Fudge LJ, Paulus M (2009). New insights into symptoms and neurocircuit function of anorexia nervosa. Nat Rev Neurosci.

[28] Kaye WH, Wierenga CE, Bailer UF, Simmons AN, Bischoff-Grethe A (2013). Nothing tastes as good as skinny feels: the neurobiology of anorexia nervosa. Trends Neurosci.

[29] Kelly C, Toro R, Di Martino A, Cox CL, Bellec P, Castellanos FX, Milham MP (2012). A convergent functional architecture of the insula emerges across imaging modalities. Neuroimage.

[30] Keramati M, Gutkin B (2014). Homeostatic reinforcement learning for integrating reward collection and physiological stability. Elife.

[31] Kim KR, Ku J, Lee J-H, Lee H, Jung Y-C (2012). Functional and effective connectivity of anterior insula in anorexia nervosa and bulimia nervosa. Neurosci Lett.

[32] Kim S-I (2013). Neuroscientific model of motivational process. Front Psychol.

[33] Kirk KM, Martin FC, Mao A, Parker R, Maguire S, Thornton LM, Zhu G, McAloney K, Freeman JL, Hay P, Madden S, Morgan C, Russell J, Sawyer SM, Hughes EK, Fairweather-Schmidt AK, Fursland A, McCormack J, Wagg F, Jordan J, Kennedy MA, Ward W, Wade TD, Bulik CM, Martin NG (2017). The Anorexia Nervosa genetics initiative: study description and sample characteristics of the Australian and New Zealand arm. Aust N Z J Psychiatry.

[34] Klabunde M, Collado D, Bohon C (2017). An interoceptive model of bulimia nervosa: a neurobiological systematic review. J Psychiatr Res.

[35] Laird AR, Robinson JL, McMillan KM, Tordesillas-Gutiérrez D, Moran ST, Gonzales SM, Ray KL, Franklin C, Glahn DC, Fox PT, Lancaster JL (2010). Comparison of the disparity between Talairach and MNI coordinates in functional neuroimaging data: validation of the lancaster transform. Neuroimage.

[36] Lancaster JL, Tordesillas-Gutiérrez D, Martinez M, Salinas F, Evans A, Zilles K, Mazziotta JC, Fox PT (2007). Bias between MNI and Talairach coordinates analyzed using the ICBM-152 brain template. Hum Brain Mapp.

[37] Lee JE, Namkoong K, Jung YC (2017). Jun 9). Impaired prefrontal cognitive control over interference by food images in binge-eating disorder and bulimia nervosa. Neurosci Lett.

[38] Lloyd EC, Joanna ES (2018). What can food-image tasks teach us about anorexia nervosa? a systematic review. J Eat Disord.

[39] Lock J, Garrett A, Beenhakker J, Reiss AL (2011). Aberrant Brain Activation during a response inhibition task in adolescent eating disorder subtypes. Am J Psychiatry.

[40] Marsh R, Horga G, Wang Z, Wang P, Klahr KW, Berner LA, Walsh BT, Peterson BS (2011). An fMRI study of self-regulatory control and conflict resolution in adolescents with bulimia nervosa. Am J Psychiatry.

[41] Miller EK, Cohen JD (2001). An integrative theory of prefrontal cortex function. Annu Rev Neurosci.

[42] Moher D, Liberati A, Tetzlaff J, Altman DG (2009). Preferred reporting items for systematic reviews and meta-analyses: the PRISMA Statement. PLoS Med.

[43] Monteleone AM, Castellini G, Volpe U, Ricca V, Lelli L, Monteleone P, Maj M (2018). Neuroendocrinology and brain imaging of reward in eating disorders: a possible key to the treatment of anorexia nervosa and bulimia nervosa. Prog Neuropsychopharmacol Biol Psychiatry.

[44] O’Hara CB, Campbell IC, Schmidt U (2015). A reward-centred model of anorexia nervosa: a focussed narrative review of the neurological and psychophysiological literature. Neurosci Biobehav Rev.

[45] Murphy FC, Nimmo-Smith I, Lawrence AD (2003). Functional neuroanatomy of emotions: a meta-analysis. Cogn Affect Behav Neurosci.

[46] Patel RS, Olten B, Patel P, Shah K, Mansuri Z (2018). Hospitalization outcomes and comorbidities of bulimia nervosa: a nationwide inpatient study. Curēus.

[47] Phillips ML, Drevets WC, Rauch SL, Lane R (2003). Neurobiology of emotion perception I: the neural basis of normal emotion perception. Biolog Psychiatry(1969).

[48] Radua J, Mataix-Cols D (2009). Voxel-wise meta-analysis of grey matter changes in obsessive-compulsive disorder. British J Psychiatry: J Mental Sci.

[49] Radua J, Mataix-Cols D, Phillips ML, El-Hage W, Kronhaus DM, Cardoner N, Surguladze S (2012). A new meta-analytic method for neuroimaging studies that combines reported peak coordinates and statistical parametric maps. European Psychiatry: J Assoc of European Psychiatrists.

[50] Radua J, Rubia K, Canales-Rodríguez EJ, Pomarol-Clotet E, Fusar-Poli P, Mataix-Cols D (2014). Anisotropic kernels for coordinate-based meta-analyses of neuroimaging studies. Front Psych.

[51] Samartsidis P, Montagna S, Nichols TE, Johnson TD (2017). The coordinate-based meta-analysis of neuroimaging data. Stat Sci: Rev J Inst Math Stat.

[52] Santel S, Baving L, Krauel K, Münte TF, Rotte M (2006). Hunger and satiety in anorexia nervosa: fMRI during cognitive processing of food pictures. Brain Res.

[53] Scaife JC, Godier LR, Reinecke A, Harmer CJ, Park RJ (2016). Differential activation of the frontal pole to high vs low calorie foods: the neural basis of food preference in Anorexia Nervosa? Psychiatry research. Neuroimaging.

[54] Schienle A, Schäfer A, Hermann A, Vaitl D (2009). Binge-eating disorder: reward sensitivity and brain activation to images of food. Biol Psychiat.

[55] Simon JJ, Stopyra MA, Friederich H-C (2019). Neural processing of disorder- related stimuli in patients with anorexia nervosa: a narrative review of brain imaging studies. J Clin Med.

[56] Steward T, Menchon JM, Jiménez-Murcia S, Soriano-Mas C, Fernandez-Aranda F (2018). Neural network alterations across eating disorders: a narrative review of fMRI studies. Curr Neuropharmacol.

[57] Tan Y, Wei D, Zhang M, Yang J, Jelinčić V, Qiu J (2018). The role of mid-insula in the relationship between cardiac interoceptive attention and anxiety: evidence from an fMRI study. Sci Rep.

[58] Turkeltaub PE, Eickhoff SB, Laird AR, Fox M, Wiener M, Fox P (2012). Minimizing within-experiment and within-group effects in activation likelihood estimation meta-analyses. Hum Brain Mapp.

[59] Uher R, Murphy T, Brammer MJ, Dalgleish T, Phillips ML, Ng VW, Andrew CM, Williams SC, Campbell IC, Treasure J (2004). Medial prefrontal cortex activity associated with symptom provocation in eating disorders. Am J Psychiatry.

[60] Van den Eynde F, Claudino AM, Mogg A, Horrell L, Stahl D, Ribeiro W, Uher R, Campbell I, Schmidt U (2010). Repetitive transcranial magnetic stimulation reduces cue-induced food craving in bulimic disorders. Biol Psychiatry(1969).

[61] Van den Eynde F, Giampietro V, Simmons A, Uher R, Andrew CM, Harvey PO, Campbell IC, Schmidt U (2013). Brain responses to body image stimuli but not food are altered in women with bulimia nervosa. BMC psychiatry.

[62] Vocks S, Busch M, Schulte D, Grönermeyer D, Herpertz S, Suchan B (2010). Effects of body image therapy on the activation of the extrastriate body area in anorexia nervosa: an fMRI study. Psychiatry Res.

[63] Wagner A, Aizenstein H, Mazurkewicz L, Fudge J, Frank GK, Putnam K, Bailer UF, Fischer L, Kaye WH (2007). Altered insula response to taste stimuli in individuals recovered from restricting-type anorexia nervosa. Neuropsychopharmacology.

[64] Walton ME, Bannerman DM, Alterescu K, Rushworth MFS (2003). Functional specialization within medial frontal cortex of the anterior cingulate for evaluating effort-related decisions. J Neurosci.

[65] Wierenga CE, Ely A, Bischoff-Grethe A, Bailer UF, Simmons AN, Kaye WH (2014). Are extremes of consumption in eating disorders related to an altered balance between reward and inhibition?. Front Behav Neurosci.

[66] Wittmann M, Lovero KL, Lane SD, Paulus MP (2010). Now or later? Striatum and insula activation to immediate versus delayed rewards. J Neurosci Psychol Econ.

[67] Wu M, Hartmann M, Skunde M, Herzog W, Friederich H-C (2013). Inhibitory control in bulimic-type eating disorders: a systematic review and meta-analysis. PLoS ONE.

[68] Young KS, Rennalls SJ, Leppanen J, Mataix-Cols D, Simmons A, Suda M, Campbell IC, O'Daly O, Cardi V (2020). Exposure to food in anorexia nervosa and brain correlates of food-related anxiety: findings from a pilot study. J Affect Disord.

[69] Zhu J-N, Wang J-J (2007). The cerebellum in feeding control: possible function and mechanism. Cell Mol Neurobiol.

